# Sex as a Biological Variable in Emergency Medicine Research and Clinical Practice: A Brief Narrative Review

**DOI:** 10.5811/westjem.2017.8.34997

**Published:** 2017-10-06

**Authors:** Alyson J. McGregor, Gillian A. Beauchamp, Charles R. Wira, Sarah M. Perman, Basmah Safdar

**Affiliations:** * Warren Alpert Medical School of Brown University, Department of Emergency Medicine, Division of Sex and Gender in Emergency Medicine, Providence, Rhode Island; † Oregon Health & Science University, Department of Emergency Medicine, Portland, Oregon; ‡ Yale University School of Medicine, Department of Emergency Medicine, New Haven, Connecticut; § University of Colorado, School of Medicine, Department of Emergency Medicine, Aurora, Colorado

## Abstract

The National Institutes of Health recently highlighted the significant role of sex as a biological variable (SABV) in research design, outcome and reproducibility, mandating that this variable be accounted for in all its funded research studies. This move has resulted in a rapidly increasing body of literature on SABV with important implications for changing the clinical practice of emergency medicine (EM). Translation of this new knowledge to the bedside requires an understanding of how sex-based research will ultimately impact patient care. We use three case-based scenarios in acute myocardial infarction, acute ischemic stroke and important considerations in pharmacologic therapy administration to highlight available data on SABV in evidence-based research to provide the EM community with an important foundation for future integration of patient sex in the delivery of emergency care as gaps in research are filled.

## INTRODUCTION

Integration of potential sex effects into biomedical research is now a requirement. The U.S. National Institutes of Health (NIH) implemented a policy [NOT-OD-15-102] expecting research design, analysis and reporting to account for the role of sex as a biological variable (SABV) in vertebrate and human studies.

In anticipation of this, emergency medicine (EM) researchers developed consensus on a sex and gender specific agenda that would guide research in emergency care for the next decade. The proceedings demonstrated the expanding influence that sex (sex chromosomes XX or XY) and gender (psycho-social identity) have on disease presentation, performance of diagnostic testing, treatment responses and outcomes.^1^ Additionally, provider behavior, healthcare utilization and disparities in delivery of medical care were also demonstrated to have effects linked to patient sex and gender.^1^,^2^

As the field of EM continues to align itself with national research priorities, new data continues to surface supporting significant differences of SABV in patient presentation, diagnostic workup, treatment and outcomes. This is a narrative review on the effect of SABV on three commonly seen EM conditions: acute myocardial infarction (AMI), acute ischemic stroke (AIS) and general administration of pharmacologic preparations. These examples are provided to briefly introduce the EM clinician and researcher to a sampling of the available SABV data, as well as to highlight the many gaps in knowledge that will need to be filled by future research endeavors in SABV in emergency care. Additionally, we discuss the cognitive steps required to bridge the gap between discovering the ever-increasing evidence that will result as SABV continues to be incorporated into EM research and the understanding of how to use this data to impact the clinical care of patients.

Due to the evolving terminology that now exists within the continuum of sex and gender, we have provided current definitions as an Appendix. Further discussion of the social construct of gender as well as the health-related issues of transgender patients are beyond the scope of this manuscript.

### Cognitive Integration of Sex as A Biological Variable (SABV) by the Practicing Emergency Provider

Emergency physicians (EP) will need to learn how to incorporate evidence where SABV may affect clinical manifestation of disease as well as diagnostic and treatment decisions. Table 1 summarizes the cognitive steps EPs can use to assist the integration of this evidence depending upon the patient’s sex. The most important first step toward integration and clinical care is to accurately identify each patient’s biological sex. This may require clarification by the patient, as it does not always follow stereotypical norms, and the chromosomal sex (XX vs. XY) may be distinct from gender identity.

After performing the appropriate focused physical exam and assessment, it is important to recognize how the patient’s sex may affect the clinical manifestation and presentation of the current illness. Routinely asking yourself, “How would the presentation, diagnostic workup or management of this patient change if they were the opposite sex?” is an important cognitive step in acknowledging this new science and aids in training ourselves to think differently.

When considering diagnostic workup, consider the potential limitations that may exist depending on patient sex. For instance, women are more likely to have non-obstructive cardiovascular disease compared to men.^3^ Many of the diagnostic tools used to detect cardiovascular disease in the emergent setting have been based upon detection of macrovascular / obstructive coronary artery disease (CAD). This will leave microvascular / non-obstructive disease or a non-plaque mediated cause of ischemia often undetectable. These limitations should be understood and discussed with the patient.

As research that includes SABV continues, more sex-specific thresholds for biomarkers and laboratory value references will become increasingly available. For instance, sex-specific thresholds now exist for troponin.^4^ Sex-specific laboratory value reference ranges are available for hemoglobin/hematocrit, calcium, creatinine, cholesterol and uric acid. The patient’s biological sex, gender, and gender identity will need to be considered when interpreting these references ranges regarding sex-specific norms. Knowledge and accurate utilization of these ranges will increasingly become important.

Pharmacokinetics and pharmacodynamics are now known to be significantly different between women and men and have been demonstrated for many drugs including zolpidem,^5^ propofol,^6^ and rocuronium.^7^ Additionally, indications for initiating medications can also differ by sex.^8^ It is crucial that EPs are aware of these differences and remain open to new data as it is published to minimize risk and optimize benefits in the use of medications in the emergency setting.

To aid the emergency care provider in consuming newly released data, as well as achieving the aims of integration of SABV into clinical practice, an effective literature-search strategy is critical. The Texas Tech University Health Sciences Center Sex and Gender Specific Health PubMed Search Tool may help facilitate SABV-based literature assessment as well as clinical decision-making.^9^

## ACUTE MYOCARDIAL INFARCTION

Case: A 40-year-old female with obesity, hypertension and one-pack-a-day smoking history was brought to the emergency department (ED) by ambulance for substernal chest discomfort at rest. Electrocardiogram (ECG) showed normal sinus rhythm with new T-wave inversions in I and AVL. Troponin came back at 0.10 (normal <0.05). Your diagnosis is non-ST elevation myocardial infarction (NSTEMI).

AMI is more common in men at a young age. However, young women with AMI are two times more likely than men to die during the hospitalization.^22^ This sex-specific mortality disadvantage persists for women 30 days post discharge, and up to five years after the MI.^23^ Some attribute this disparity to higher prevalence of comorbidities in women with AMI while others point to less aggressive management of women.^24^,^25^ However, neither entirely explains the higher mortality seen in women – a cohort typically protected by estrogen. When MI occurs despite this protection, it is due to different disease pathology, anatomical differences or variable mechanisms of atherosclerosis.^26^–^28^

Table 2 outlines some of the sex differences in the pathophysiology of ischemic syndromes. One in eight women with AMI have a pathophysiology other than the classic thrombo-embolic mechanism that underlies typical AMI.^27^ Women are also more likely to have single vessel disease (resulting in higher rates of false-negative stress tests).^29^ Even in the presence of an abnormal stress test or positive troponin, women are less likely to have >50% obstruction of the coronary artery, a *sine qua non* of heart disease.^3^,^26^ Ischemia in absence of CAD is most commonly attributed to coronary microvascular dysfunction (CMD). It refers to a group of mechanisms causing endothelial dysfunction of the small vessels (arterioles and prearterioles) that limit the ability of myocardium to increase blood flow in response to stress (coronary flow reserve) resulting in ischemic chest pain.

CMD is five times more common in women and presents a diagnostic challenge as routine testing for obstructive CAD is not sensitive in diagnosing CMD.^30^–^32^ The EP often encounters these alternate mechanisms of ischemia while evaluating patients with recurrent chest pain, one of the leading causes of readmissions.^33^ CMD is a known cause of recurrent chest pain and needs to be considered in the workup of patients with chest pain who are ruled out for CAD.^34^–^35^

Differences in pathophysiology also influence the presentation and risk stratification of AMI. Women, like men, present most commonly with chest pain when having an AMI.^36^ However, they describe it more as *discomfort* than *pain* and are less likely to describe it as “classic” angina, i.e., as exertional substernal chest pain, which is more reliably predictive of obstructive CAD in men.^37^ What also distinguishes the presentation is the number of associated symptoms: Women tend to describe a *cluster* of symptoms such as shortness of breath, fatigue, and radiation of pain, *in addition* to chest discomfort as opposed to men who focus primarily on chest pain.^38^ The cluster of symptoms could therefore “dilute” the presentation when evaluating women with ischemia compared to men. This was seen in a sex-specific study of AMI from Canada,^38^ and summarized in a recent consensus statement on AMI.^23^ While more commonly seen in women, atypical symptoms such as shortness of breath, epigastric pain, nausea as the sole presentation of AMI constitute only 10–15% of all STEMI presentations and less than a third of all AMI presentations.^39^,^40^ Young women with AMI may also present without any chest pain compared to men, potentially explaining the higher mortality seen in this age group.^41^

While assessment of cardiac risk factors in the ED has been questioned, data supports their use for cardiovascular risk stratification particularly in young patients.^42^,^43^ Certain risk factors impart a differential risk by sex. Examples are included in Table 1 but the list of novel risk factors is rapidly evolving. The EP may inquire specifically about these risk factors based on patient sex and incorporate them in assigning the pretest probability for ischemia for that patient. For instance, the presence of diabetes in a young patient with chest pain would warrant some caution as diabetes has been known to equalize the cardiovascular risk protection typically seen in young women compared to similarly aged men.^44^,^45^

Several risk stratification tools currently exist to aid the EP in evaluating patients with chest pain. The more commonly used scores include the Thrombolysis in Myocardial Infarction (TIMI) score, the HEART (History, ECG, Age, Risk factors, Troponin) score or the Emergency Department Assessment of Chest Pain Score [EDACS]; all aim at identifying low-risk patients who could be safely discharged early from the ED.^46^ While most of these scores considered sex/gender as a control variable in the original derivation cohorts, not many validated their performance independently among men and women. In the few studies that did evaluate these scores by sex, differential performance was seen despite similar discriminatory and calibration characteristics^47^ of the scores.^48^ Men were noted to have worse outcomes, suggesting that early discharge for low-risk men by HEART or TIMI scores may be less safe for men as compared to women with acute chest pain. This is likely due to the higher rates of major adverse outcomes (MACE) seen in men as compared to women with AMI, highlighting the need for sex-specific risk stratification scores.^49^ The differential effect of sex was considered in the EDACS in its final model.^46^ It performed well in its original validation cohort with 100% sensitivity and 59% specificity for low-risk patients. While the EDACS is a step forward by incorporating patient sex in considering cardiac risk, it still needs validation in studies evaluating its performance independently by sex.^49^

SABV can influence the interpretation of diagnostic tests for AMI.^11^ Troponin testing is the cornerstone of diagnosing AMI in the ED. A European study of 1,126 patients with acute coronary syndrome (55% men and 45% women) used a conventional troponin assay to diagnose 19% men and 11% women with AMI.^11^ The use of a high-sensitivity troponin assay added 4–5% AMI cases to each group. Most interestingly, when using a sex-specific threshold for troponin that has a lower cut-off for women (given their lower heart muscle mass), the diagnosis of MI doubled in women and did not vary significantly for men. Patients who were reclassified using the sex-specific cut-off had worse outcomes than patients with conventionally diagnosed MI.^11^ High-sensitivity troponin assays were only recently approved by the FDA for use in the U.S. and therefore need validation in U.S. populations. However, the results are intriguing and may influence the future of cardiac marker analysis.^50^

Interpretation of stress tests may also vary depending on whether the patient is a man or a woman. For instance, an exercise tolerance test has 60% sensitivity in women (often with single vessel disease) as opposed to 71% sensitivity in men (often with multi-vessel disease).^10^ Women are also less likely to achieve maximal heart rate crucial for correct interpretation and more likely to be converted to pharmacological testing. Sensitivity for a pharmacological nuclear scan in general is less (60%) compared to an exercise perfusion scan (85%).^10^ Hence, women are more likely to have tests with lower sensitivity than men. Additional considerations, such as radiation risk in breast tissue with computed tomography (CT) angiography, further influence the optimal choice of test for men vs. women.

With a rapidly growing body of science on ischemic syndromes beyond CAD, an EP will be tasked to incorporate this knowledge into his/her assessment of chest pain patients to capture all cases of cardiac ischemia. Diagnosing non-CAD causes of ischemia can, however, be challenging. In contrast to CAD, CMD can present as angina induced by atypical triggers such as change in temperature or high stress.^34^,^51^–^53^ Also, conventional tests such as troponin or stress test may be normal with CMD.^31^ Our preliminary work indicates that up to 40% of ED patients with recurrent chest pain have CMD by advanced imaging.^34^ Patients are suspected for CMD based on recurrent symptoms consistent with angina and lack of evidence for obstructive CAD. An EP may refer such patients for additional testing such as cardiac positron emission tomography (PET), magnetic resonance imaging (MRI) or even coronary reactivity testing by coronary angiogram so a definitive diagnosis of ischemia may be made.^51^

Current guidelines for treating MI are similar for men and women including benefits of anticoagulants, with some sex-specific nuances. For NSTEMI due to CAD, early invasive therapy is harmful for low-risk women and beneficial for all-risk men.^54^ In addition, anticoagulants have to be weight-adjusted to reduce the risk of bleeding, more often seen in women.^21^ For STEMI, newer therapies based on plaque composition such as aspiration thrombectomy and catheter-directed lytic therapy without implantation of stents are on the horizon.^23^ A small study of these non-stent therapies indicates that the outcomes may be comparable to conventional therapies.^55^ The biggest changes on the horizon are with regard to the treatment guidelines for non-CAD related causes of ischemia. Revascularization is not indicated for some causes of AMI (such as coronary artery dissection) or applicable (coronary artery vasospasm). Additional therapies may need to be adapted to the underlying cause, for instance calcium channel blockers for vasospasm, early use of statins and ACE inhibitors for non-plaque AMI,^56^ or no anticoagulants for coronary artery dissection. For treatment of CMD and recurrent angina, at minimum patients require cardioprotective medications such as aspirin, statins and an aggressive symptom-management plan that may include ACE-inhibitors and statins, while nitrates and beta-blockers appear ineffective.^57^ Given the effect of SABV in rapidly changing the landscape of ischemic heart disease, this review is meant to serve as a primer for a practicing physician.

## ACUTE ISCHEMIC STROKE

Case: A 39-year-old female two weeks post-Cesarean section with rheumatic heart disease and paroxysmal atrial fibrillation (anticoagulation held) developed an acute right MCA stroke syndrome (NIH Stroke Scale (NIHSS) score of 19; left side flaccid and plegic in arm and leg). Intravenous (IV) tissue plasminogen activator (TPA) had no improvement; the patient underwent mechanical thrombectomy 5.5 hours after symptom onset. A week later symptoms had nearly resolved.

Women face a higher lifetime risk of stroke having ~55,000 more strokes than men each year,^66^ and have a higher annual stroke death rate (fifth cause of death for men; fourth for women). Overall, when age adjusted the incidence of all cerebrovascular disease is higher in men than women at younger ages but not older ages.^67^ From 1980 to 2005 there was a greater age-adjusted decline in stroke death rates in men than in women.^67^ There are roughly 6.8 million stroke survivors in the United States living after having had a stroke, including 3.8 million women and 3 million men.^21^ Overall, one in five women will have a stroke at some point in their lives.^68^ Regarding strokes in young people—in some studies men appear to have a higher rate in the 35- to 44-year-old age group, while other population-based analyses reveal a higher incidence among women under 30 years old.^70^

Sex-specific considerations in AIS are summarized in Table 3. Although the absolute risk of stroke is low, there are several sex-specific risk factors unique to women that increase the relative risk of stroke—hormone therapy (HT), migraine headaches with aura in combination with tobacco use, and preeclampsia/eclampsia.^66^ Of note, atrial fibrillation is more common in women contrasted to men over the age of 75 while rates below this age are equivocal. The specific combination of hypertension and HT further increases the risk of stroke,^68^ and the post-partum hypercoagulability phase may now extend out to 12 weeks post-delivery.^70^ Recently, the American Stroke Association (ASA) published guidelines for prevention of stroke in women.^66^ However, there is still opportunity for future progress, including addressing sex and gender differences in other phases of stroke care.

Several key sex differences have been evaluated in secondary analyses of large registries and other investigations. Population-based data on sex differences in the acute phase of presentation indicate conflicting results. Some studies report that women have a higher propensity for presenting with non-traditional stroke symptoms (i.e., altered mental status)^77^–^2^ contrasted to their male counterparts who have higher odds of presenting with specific symptoms (i.e., focal findings such as paresthesia, ataxia, and diplopia) in combination with non-specific symptoms (i.e., pain, disorientation, generalized weakness, fatigue).^73^–^4^ Others studies have not observed a consistent sex difference in the prevalence of traditional vs. non-traditional symptoms “making it difficult to craft a public health message about gender differences in early warning signs of stroke.”^75^ It is possible that the above investigations may be prone to length bias due to delayed presentations to the ED. One study observed that the risk of delay in hospital arrival was three times greater for women with acute strokes contrasted to men.^76^ However, other literature, while not focusing overall on time to presentation, has demonstrated women are more likely to arrive via ambulance compared to men^77^ while being less likely to receive TPA. Further investigation in this area is necessary to evaluate these differences.

For stroke etiology, some studies suggest women may be more likely to have large territory strokes contrasted to lacunar/small embolic syndromes more prevalent in men.^78^ But interpretation is limited as other studies posit that women are more likely to have cardioembolic strokes contrasted to men.^75^ Such studies do not specifically differentiate risks in different age categories as the etiology of stroke varies in young to geriatric patients. To further complicate matters for the EP, patients presenting with stroke mimics, such as complex migraine tend to be younger and are more likely to be female.^79^–^80^ Navigating this paradox—the simultaneous existence of atypical presentations and observed differences in treatment rates—is a difficult task for the EP. One solution is to have a system that can rapidly recruit the aid of a stroke specialist (i.e., via telemedicine) and in some cases perform definitive diagnostic testing (i.e., hyperacute MRI) in cases where diagnostic uncertainty exists. While the current literature does not demonstrate sex differences in the rates of missed strokes, further investigation in this area merits consideration.^81^

A concerning observation as cited above is that women are less likely to receive IV TPA. This could be especially deleterious to outcomes, as other pooled analyses of clinical trials suggest a larger treatment effect of TPA in women including a NINDS/ATLANTIS/ECASS II pooled analysis,^82^ and PROACT-2.^83^ Of note, among patients receiving placebo, the former pooled analysis also observed worse outcomes in women compared to men. While these differences may partially be explained by factors such as age, initial NIHSS, delays in presentation, co-morbidities, the possibility of a stroke mimic, and the possible association of non-traditional symptoms, future investigation is necessary.^84^ Some studies have found that sex differences were equalized after treatment.^85^ In this new era of mechanical thrombectomy there is limited knowledge regarding sex differences; however, it is known that women are less likely to undergo cerebral angiography^86^ and that women have smaller diameter cerebral vessels.^87^ The influence on patient selection and treatment effect is unknown.

For stroke systems research, a national registry analysis revealed women are less likely than their male counterparts to receive defect-free care across the age spectrum. Defect-free care is defined as a health system attaining all of the AHA/ASA quality benchmarks in different phases of stroke care (i.e., consideration of IV TPA, anticoagulation for atrial fibrillation, dysphagia screening, etc).^88^ The magnitude of such observations on overall clinical outcomes is unknown. However, other studies evaluating outcomes reveal women are more likely to have poor functional outcomes after AIS at 90 days and one year, and are institutionalized more often compared with men.^84^

In pregnancy, AIS is rare with an overall incidence ranging from 9–34 per 100,000^89^ and has neither randomized controlled data nor registry data to inform decisions, given this population is commonly excluded from trials. Management requires a systems approach involving providers from multiple disciplines (i.e., high-risk obstetrics, neurology), and typically relies upon extrapolation from existing guidelines. For eligible patients meeting criteria for thrombolytic therapy risks and benefits should be evaluated on a case-by-case basis with the involvement of high-risk obstetrics and neurology, as well as the patient and family.^90^ Endovascular therapy merits strong consideration for patients with large vessel occlusions and is potentially the most optimal intervention per some stroke experts.^91^

### Sex-Based Pharmacology Considerations in the Emergency Department

Case: A 58-year-old male trauma patient presents to the ED. The EP notes he requires analgesia, antiemetics, procedural sedation, rapid sequence intubation and vasopressor support.

The provider should be aware of the available data on sex-based dosing of analgesia, antiemetics, sedation medications, neuromuscular blockade, vasopressors, and inotropes. The available research in SABV in response to pharmacologic therapies used in EM is limited to basic science studies, animal studies, and limited clinical studies, with a remarkable paucity of studies focused on clinical EM. Further research is needed to elucidate next steps in the translation of available data into safe and effective clinical practice.

As in the management of this trauma patient, EPs frequently manage patients with pharmacotherapies. Physiological, hormonal, and genetic differences between male and female patients affect both drug response and rate of adverse drug reactions (ADR), with female patients experiencing higher drug concentrations and more ADRs, even after accounting for weight-based dosing.^28^,^92^–^7^ Examples of sex-based differences in response to pharmacologic therapies in the ED are summarized in Table 4. Historically, females have been excluded from pharmaceutical development research and sex-based dosing recommendations are rarely made.^12^,^28^ Sex alters both pharmacokinetics and pharmacodynamics.^95^,^98^ Sex differences have been reported in plasma volume, protein concentration, gastric emptying and gut transit times, drug metabolism and clearance, drug transporter function, drug receptor concentration and rates of polypharmacy.^28^,^92^,^95^,^98^–^102^

EPs providing opioid pain control should note that morphine causes lower rates of respiratory depression and has more rapid onset and offset in males.^12^–^13^,^103^ Female patients are more sensitive to pain in experimentally-induced pain studies; have increased willingness to report pain in studies of pain tolerance; have higher rates of chronic painful conditions in epidemiological studies studying pain in multiple body sites; and may require higher doses of opioids to obtain pain relief, which may place female patients at increased risk for adverse events, particularly given an increased risk of opioid-induced respiratory depression compared to males.^12^–^15^,^104^ Given the available data, careful dose titration and close monitoring for both effectiveness and opioid-induced side effects is warranted even when using standard weight-based dosing of morphine. In the case above, the provider may note that male patients may be able to tolerate higher doses of morphine, may be less likely to report pain, and are at decreased risk for adverse effects from opioids compared to females.

Underlying QTc prolongation and drug-induced torsades de pointes (TdP) from many antiemetics, antipsychotics, antiarrhythmics, and antimicrobials commonly administered in the ED is more common in females and may vary within the menstrual cycle.^105^–^8^ Underlying mechanisms may be related to protective effects of testosterone in males, pro-arrhythmic effects of estrogens in females, and sex-based differences in drug pharmacokinetics and ion channel expression.^92^,^105^–^6^,^109^–^10^ As an example, if this male trauma patient is treated with the antiemetic ondansetron, baseline QTc prolongation and therefore drug-induced TdP is less likely than in a female patient. A baseline ECG to assess QTc interval could be performed prior to administering QTc prolonging medications in patients at higher risk for TdP, such as female patients, patients with congenital long QT, or patients with concomitant use of another QT-prolonging medication.^109^

Given the same dose of a neuromuscular blocking agent like rocuronium or vecuronium, male patients will have a lower serum drug concentration, slower onset, and shorter duration of action compared to females.^7^,^12^ In the case of this male patient, the provider may note that paralytic effect during rapid sequence intubation may be shorter in duration than it would be for a female patient receiving standard doses of rocuronium. Such sex differences have not been found with succinylcholine.

When serial bolus dosing of propofol is used to titrate conscious sedation to effect, male patients require lower and fewer doses of propofol to maintain the same blood concentrations of drug as female patients, and often wake up more slowly than female patients.^17^–^19^ Percentage body fat is higher in females, which lowers the blood concentration of lipid soluble drugs such as propofol.^89^ Should this male patient require deep sedation using propofol, the EP may note that the patient may wake up more slowly, and require lower doses within the standard dosing range for propofol when compared to a female patient.^28^

Male patients metabolize vasopressor and inotropic agents commonly administered in the ED such as norepinephrine, epinephrine, and dopamine at higher rates than female patients due to 25% higher catabolic enzyme activity.^12^,^20^ If the resuscitation of this male trauma patient warrants the titration of a vasopressor or inotrope to effect, the clinician may find that this male patient requires doses on the higher end of the normal dosing range for a vasopressor or inotrope.

Given that many of the medications provided in the ED are already dosed in ranges and titrated to effect, providers should be aware that female patients may be more susceptible to side effects from standard dosing of morphine; more sensitive to standard doses of inotropes, vasopressors, and non-depolarizing paralytics such as rocuronium; and less sensitive to standard doses of propofol than male patients. An awareness of these differences that have been reported in the literature can help clinicians be cognizant of sex-based differences that may occur in response to medication administration within dosing ranges commonly used within the standard practice of EM. Finally, given these sex-based differences in drug response and adverse outcomes, SABV should be considered in all phases of the development of new drugs designed to be used in the ED.^92^,^106^

## LIMITATIONS

Despite the importance of both variables, sex and gender, in research and the important considerations they impart in the clinical care of women and men, this brief narrative review focused on SABV in research as it relates to ED clinical care. The ability to accurately measure and access the impact of gender identity on the delivery of medical care is complex and currently without well agreed-upon validated measurement tools. Despite this, the authors feel it is important to initially assess each patient’s biological sex and current gender identity, as it will begin the process of realizing the fact that these two variables may not always be congruent.

The authors also discovered a paucity of SABV being included within EM clinical practice guidelines and available research, highlighting this as an area for future research and inclusion.

## CONCLUSION

With the new NIH requirement to integrate SABV into research design, analysis and reporting, the evidence for the role of patient sex in medical care is growing. As these three clinical scenarios demonstrate, evidence for incorporating SABV in clinical care has immediate implications for the ED patient. The cognitive steps outlined in the manuscript are designed to prepare EM clinicians to identify their patients based upon biological sex, and to become more cognizant of sex-based variability in presentation of disease, appropriateness of diagnostic testing and interpretation of results. Additionally, better incorporation of SABV will allow for safe and effective sex-based treatment in EM. An awareness of known SABV factors in the field of EM will prepare the clinician to consume emerging data in this area. We encourage the utilization of the validated PubMed search tool described to assist the emergency physician in extracting new evidence through the lens of this important biological variable.

Translation of new knowledge of SABV to the bedside requires an awareness of both the available literature and the knowledge gaps that exist in understanding of how sex-based research will ultimately impact patient care. This manuscript details three specific clinical scenarios in which sex based care is imperative, however, much information is still needed to better dissect the role of SABV in all aspects of emergency care. It is incumbent on the astute emergency care provider to consciously embrace emerging evidence in order to provide the best clinical care to their patients. Sex is not simply a descriptor, but a biologic variable that has consequences on risk stratification, utilization of diagnostic tests, and appropriateness of pharmacologics ordered.

## Supplementary Information



## Figures and Tables

**Table 1:** Cognitive steps to integrate SABV* into clinical practice with associated examples from current literature.

Cognitive Step	Examples
1. Identify patient sex	Male or Female
2. Understand sex differences in clinical manifestation of disease	Females more likely to have coronary microvascular disease than men^3^
3. Recognize potential limitations in diagnostic testing	Variable prognosis of exercise treadmill test in man versus woman^10^
4. Use any sex-specific thresholds for biomarkers or laboratory value references	Troponin,^11^ Hemoglobin/Hematocrit, Calcium, Creatinine, Cholesterol and Uric Acid
5. When available, dose medications based upon sex-specific evidence	Sex-based dosing of analgesia,^12^–^15^ antiemetics,^16^ sedation medications,^17^–^19^ neuromuscular blockade,^17^,^12^ vasopressors or inotropes,^12^,^20^, anticoagulants for treatment of myocardial infarction^21^
6. Use Sex and Gender Specific Health PubMed Search Tool	www.sexandgenderhealth.org

**Table 2:** Sex differences in pathophysiology of cardiac ischemic syndromes.

Acute coronary syndromes	Women	Men
Anatomical area of coronary obstruction	Large and small vessels	Large vessels
Pathophysiology of ischemia	Plaque rupture	Plaque rupture^58^
	Plaque erosion	
	Alternate mechanisms: 23	
	coronary artery spasm	
	spontaneous coronary artery dissection embolization,	
	coronary microvascular dysfunction	
Presentation	Chest pain most common	Chest pain most common
	Often with cluster of associated symptoms^38^	Fewer associated symptoms
Risk factors		
Traditional		
Smoking	2-fold higher,^59^	
Diabetes	2-fold higher^60^	
Hypertension	60% higher^43^	Higher^23^
Physical inactivity	Higher	
Novel risk factors	60% higher	
Depression	Higher^61^	
Lupus		
Hypercoagulable states		
Metabolic syndrome		
Pregnancy associated	Preeclampsia gestational diabetes, preterm labor and neonatal death double the risk for AMI. 62–5	Not applicable
Diagnosis		
Troponin	Higher-sensitivity troponin using sex-adjusted cut-offs picked up twice as many with worse outcomes.^11^	High-sensitivity troponin made no significant change for diagnosing AMI compared to conventional troponin
Risk scores		Men with low-risk HEART and TIMI score have worse outcomes.^47^,^48^
Stress imaging	For intermediate-high risk, sensitivity can be increased with stress echo or nuclear stress with imaging.	ETT has higher sensitivity
Management		
STEMI	PCI preferred over thrombolytic therapy Door-to-balloon times longer^21^	PCI preferred over thrombolytic therapy
NSTEMI	Similar benefit Lower dose of anticoagulants	Similar benefit
Non-CAD ischemia	Conservative management with secondary prevention	
Prognosis		
Mortality	Higher at younger age^22^	Higher overall
Readmissions for chest pain	Higher	

*AMI*, acute myocardial infarction; *HEART*, HeartScore is a cardiovascular disease risk assessment and management tool developed by the European Society of Cardiology; *TIMI*, Thrombolytics in Myocardial Infarction, *ETT*, exercise tolerance test; *PCI*, percutaneous coronary intervention.

**Table 3:** Sex-specific considerations in acute ischemic stroke unique to women.

Risk Factors 66,68,70
Hormonal therapy 66,68
Migraines with aura in combination with tobacco 66
Preeclampsia/eclampsia 66
Peri-partum hypercoagulability 70
Atrial fibrillation (incidence higher in women when age >75 y.o.) 66
Presentation/diagnosis 71–4, 77, 79–80, 90
Possible propensity for presenting with non-traditional symptoms 71–4
Higher population incidence of conditions which may cause stroke mimic (i.e.: complex migraine, conversion disorder) 79–80
More likely to arrive to emergency department via ambulance 77
May have MRI rather than CT imaging (i.e.: pregnancy, young age) 90
Treatment 78, 82, 83, 87
Less likely to receive thrombolytic therapy 77
Potential greater treatment effect of thrombolytic therapy in women 82, 83
Smaller diameter vessels potentially effecting distal vessel thombectomy^87^

*MRI*, magnetic resonance imaging; *CT*, computed tomography.

**Table 4:** Examples of sex-based differences in response to pharmacologic therapies in the emergency department.

Response	Example
Pharmacokinetic differences^12^,^95^–^6^,^98^	Female patients may require higher doses of lipophilic medications like propofol^17^–^19^ Female patients may require lower doses of water-soluble drugs like rocuronium^7^,^12^
Volume of distribution	
Protein binding of drugs	
Metabolism and transport of drugs	
Pharmacodynamic differences^12^,^95^–^6^,^98^	Females are at increased risk of drug-induced torsades de pointes from QTc-prolonging medications such as ondansetron^105^,^107^–^9^; Female patients have increased risk of side effects such as CNS or respiratory depression from morphine^12^–^15^,^104^
Number of receptors & receptor binding	
Variability in ion channels	

*QTc*, QT interval corrected for heart rate; *CNS*, central nervous system.

## References

[1] Greenberg MR, Safdar B, Choo EK (2014). Future directions in sex- and gender-specific emergency medicine. Acad Emerg Med.

[2] McGregor AJ, Greenberg M, Safdar B (2013). Focusing a gender lens on emergency medicine research: 2012 update. Acad Emerg Med.

[3] Reis SE, Holubkov R, Conrad Smith AJ (2001). Coronary microvascular dysfunction is highly prevalent in women with chest pain in the absence of coronary artery disease: results from the NHLBI WISE study. Am Heart J.

[4] Eggers KM, Johnston N, James S (2014). Cardiac troponin I levels in patients with non-ST-elevation acute coronary syndrome-the importance of gender. Am Heart J.

[5] Greenblatt DJ, Harmatz JS, Singh NN (2014). Gender differences in pharmacokinetics and pharmacodynamics of zolpidem following sublingual administration. J Clin Pharmacol.

[6] Hoymork SC, Raeder J (2005). Why do women wake up faster than men from propofol anaesthesia?. Br J Anaesth.

[7] Adamus M, Gabrhelik T, Marek O (2008). Influence of gender on the course of neuromuscular block following a single bolus dose of cisatracurium or rocuronium. Eur J Anaesthesiol.

[8] Berger JS, Roncaglioni MC, Avanzini F (2006). Aspirin for the primary prevention of cardiovascular events in women and men: a sex-specific meta-analysis of randomized controlled trials. JAMA.

[9] Song MM, Simonsen CK, Wilson JD (2016). Development of a PubMed Based Search Tool for Identifying Sex and Gender Specific Health Literature. J Womens Health (Larchmt).

[10] Mieres JH, Gulati M, Bairey Merz N (2014). Role of noninvasive testing in the clinical evaluation of women with suspected ischemic heart disease: a consensus statement from the American Heart Association. Circulation.

[11] Shah AS, Griffiths M, Lee KK (2015). High sensitivity cardiac troponin and the under-diagnosis of myocardial infarction in women: prospective cohort study. BMJ.

[12] Booij LH (2008). Sex, age, and genetics in anesthesia. Curr Opin Anaesthesiol.

[13] Sarton E, Olofsen E, Romberg R (2000). Sex differences in morphine analgesia: an experimental study in healthy volunteers. Anesthesiology.

[14] Cepeda MS, Carr DB (2003). Women experience more pain and require more morphine than men to achieve a similar degree of analgesia. Anesth Analg.

[15] Zubieta JK, Smith YR, Bueller JA (2002). Mu-opioid receptor-mediated antinociceptive responses differ in men and women. J Neurosci.

[16] Freedman SB, Uleryk E, Rumantir M (2014). Ondansetron and the risk of cardiac arrhythmias: a systematic review and postmarketing analysis. Ann Emerg Med.

[17] Ward DS, Norton JR, Guivarc’h PH (2002). Pharmacodynamics and pharmacokinetics of propofol in a medium-chain triglyceride emulsion. Anesthesiology.

[18] Kodaka M, Suzuki T, Maeyama A (2006). Gender differences between predicted and measured propofol C(P50) for loss of consciousness. J Clin Anesth.

[19] Hoymork SC, Raeder J (2005). Why do women wake up faster than men from propofol anaesthesia?. Br J Anaesth.

[20] Boudikova B, Szumlanski C, Maidak B (1990). Human liver catechol-O-methyltransferase pharmacogenetics. Clin Pharmacol Ther.

[21] Go AS, Mozaffarian D, Roger VL (2013). Heart disease and stroke statistics--2013 update: a report from the American Heart Association. Circulation.

[22] Vaccarino V, Parsons L, Every NR (1999). Sex-based differences in early mortality after myocardial infarction. National Registry of Myocardial Infarction 2 Participants. N Engl J Med.

[23] Mehta LS, Beckie TM, DeVon HA (2016). Acute Myocardial Infarction in Women: A Scientific Statement From the American Heart Association. Circulation.

[24] Bairey Merz CN, Shaw LJ, Reis SE (2006). Insights from the NHLBI-Sponsored Women’s Ischemia Syndrome Evaluation (WISE) Study: Part II: gender differences in presentation, diagnosis, and outcome with regard to gender-based pathophysiology of atherosclerosis and macrovascular and microvascular coronary disease. J Am Coll Cardiol.

[25] Chandra NC, Ziegelstein RC, Rogers WJ (1998). Observations of the treatment of women in the United States with myocardial infarction: a report from the National Registry of Myocardial Infarction-I. Arch Intern Med.

[26] Bairey Merz CN, Johnson BD, Sharaf BL (2003). Hypoestrogenemia of hypothalamic origin and coronary artery disease in premenopausal women: a report from the NHLBI-sponsored WISE study. J Am Coll Cardiol.

[27] Spatz ES, Curry LA, Masoudi FA (2015). The VIRGO Classification System: A Taxonomy for Young Women with Acute Myocardial Infarction. Circulation.

[28] Anderson GD (2008). Gender differences in pharmacological response. Int Rev Neurobiol.

[29] Lawesson SS, Stenestrand U, Lagerqvist B (2010). Gender perspective on risk factors, coronary lesions and long-term outcome in young patients with ST-elevation myocardial infarction. Heart.

[30] Safdar B, D’Onofrio G (2016). Women and Chest Pain: Recognizing the Different Faces of Angina in the Emergency Department. Yale J Biol Med.

[31] Sara JD, Widmer RJ, Matsuzawa Y (2015). Prevalence of Coronary Microvascular Dysfunction Among Patients With Chest Pain and Nonobstructive Coronary Artery Disease. JACC: Cardiovasc Interv.

[32] Phan A, Shufelt C, Merz CN (2009). Persistent chest pain and no obstructive coronary artery disease. JAMA.

[33] Vashi A, Fox J, Carr B (2013). Use of hospital-based acute care among patients recently discharged from the hospital. JAMA.

[34] Safdar B, D’Onofrio G (2016). Women and Chest Pain: Recognizing the Different Faces of Angina in the Emergency Department. Yale J Biol Med.

[35] Kaski J, Elliott P (1995). Angina pectoris and normal coronary arteriograms: clinical presentation and hemodynamic characteristics. Am J Cardiol.

[36] Rubini Gimenez M, Reiter M, Twerenbold R (2014). Sex-specific chest pain characteristics in the early diagnosis of acute myocardial infarction. JAMA Intern Med.

[37] Hemal K, Pagidipati NJ, Coles A (2016). Sex Differences in Demographics, Risk Factors, Presentation, and Noninvasive Testing in Stable Outpatients With Suspected Coronary Artery Disease: Insights From the PROMISE Trial. JACC Cardiovascular Imaging.

[38] Khan NA, Daskalopoulou SS, Karp I (2013). Sex differences in acute coronary syndrome symptom presentation in young patients. JAMA Intern Med.

[39] McSweeney JC, Cody M, O’Sullivan P (2003). Women’s early warning symptoms of acute myocardial infarction. Circulation.

[40] D’Onofrio G, Safdar B, Lichtman JH (2015). Sex differences in reperfusion in young patients with ST-segment-elevation myocardial infarction: results from the VIRGO study. Circulation.

[41] Canto JG, Rogers WJ, Goldberg RJ (2012). Association of age and sex with myocardial infarction symptom presentation and in-hospital mortality. JAMA.

[42] Anand SS, Islam S, Rosengren A (2008). Risk factors for myocardial infarction in women and men: insights from the INTERHEART study. Eur Heart J.

[43] Yusuf S, Hawken S, Ounpuu S (2004). Effect of potentially modifiable risk factors associated with myocardial infarction in 52 countries (the INTERHEART study): case-control study. Lancet.

[44] Kalyani RR, Lazo M, Ouyang P (2014). Sex differences in diabetes and risk of incident coronary artery disease in healthy young and middle-aged adults. Diabetes Care.

[45] Huxley R, Barzi F, Woodward M (2006). Excess risk of fatal coronary heart disease associated with diabetes in men and women: meta-analysis of 37 prospective cohort studies. BMJ.

[46] Than M, Flaws D, Sanders S (2014). Development and validation of the Emergency Department Assessment of Chest pain Score and 2 h accelerated diagnostic protocol. Emerg Med Australas.

[47] Karounos M, Chang AM, Robey JL (2007). TIMI risk score: does it work equally well in both males and females?. Emerg Med J.

[48] Bank IEM, de Hoog VC, de Kleijn DPV (2017). Sex-Based Differences in the Performance of the HEART Score in Patients presenting to the emergency department with acute chest pain. J Am Heart Assoc.

[49] Safdar B, Nagurney JT, Anise A (2014). Gender-specific research for emergency diagnosis and management of ischemic heart disease: proceedings from the 2014 Academic Emergency Medicine Consensus Conference Cardiovascular Research Workgroup. Acad Emerg Med.

[50] Wendling P US debut of ‘high sensitivity’ troponin assay signals sea change in acute MI.

[51] Task Force Members, et al. (2013). 2013 ESC guidelines on the management of stable coronary artery disease: the Task Force on the management of stable coronary artery disease of the European Society of Cardiology. Eur Heart J.

[52] Masumoto A, Mohri M, Takeshita A (2001). Three-year follow-up of the Japanese patients with microvascular angina attributable to coronary microvascular spasm. Int J Cardiol.

[53] Matsuzawa Y, Sugiyama S, Sugamura K (2010). Digital assessment of endothelial function and ischemic heart disease in women. J Am Coll Cardiol.

[54] O’Donoghue M, Boden WE, Braunwald E (2008). Early invasive vs conservative treatment strategies in women and men with unstable angina and non-ST-segment elevation myocardial infarction: a meta-analysis. JAMA.

[55] Prati F, Uemura S, Souteyrand G (2013). OCT-based diagnosis and management of STEMI associated with intact fibrous cap. JACC Cardiovasc Imaging.

[56] Lindahl B, Baron T, Erlinge D (2017). Medical therapy for secondary prevention and long-term outcome in patients with myocardial infarction with non-obstructive coronary artery (MINOCA) disease. Circulation.

[57] Marinescu MA, Loffler AI, Ouellette M, Smith L, Kramer CM, Bourque JM (2015). Coronary microvascular dysfunction, microvascular angina, and treatment strategies. JACC Cardiovasc Imaging.

[58] Patel MR, Peterson ED, Dai D (2010). Low diagnostic yield of elective coronary angiography. N Engl J Med.

[59] Prescott E, Hippe M, Schnohr P, Hein HO, Vestbo J (1998). Smoking and risk of myocardial infarction in women and men: longitudinal population study. BMJ.

[60] Huxley RR, Peters SA, Mishra GD (2015). Risk of all-cause mortality and vascular events in women versus men with type 1 diabetes: a systematic review and meta-analysis. Lancet Diabetes Endocrinol.

[61] Smolderen KG, Strait KM, Dreyer RP (2015). Depressive symptoms in younger women and men with acute myocardial infarction: insights from the VIRGO study. J Am Heart Assoc.

[62] Wenger NK, Ouyang P, Miller VM (2016). Strategies and Methods for Clinical Scientists to Study Sex-Specific Cardiovascular Health and Disease in Women. J Am Col of Cardiology.

[63] Robbins CL, Hutchings Y, Dietz PM (2014). History of preterm birth and subsequent cardiovascular disease: a systematic review. Am J Obstet Gynecol.

[64] Heida KY, Velthuis BK, Oudijk MA (2016). Dutch Guideline Development Group on Cardiovascular Risk Management after Reproductive Disorders, Cardiovascular disease risk in women with a history of spontaneous preterm delivery: A systematic review and meta-analysis. Eur J Prev Cardiol.

[65] Rich-Edwards JW, Fraser A, Lawlor DA (2014). Pregnancy characteristics and women’s future cardiovascular health: an underused opportunity to improve women’s health?. Epidemiol Rev.

[66] Bushnell C, McCullough LD, Awad IA (2014). Guidelines for the prevention of stroke in women: a statement for healthcare professionals from the American Heart Association/American Stroke Association. Stroke.

[67] Lloyd-Jones D, Adams R, Carnethon M (2009). Heart disease and stroke statistics—2009 update: a report from the AHA statistics committee and stroke statistics subcommittee. Circulation.

[68] Association, A. S. Women Face Higher Risk of Stroke. New Guideline Offeres Ways to Lower Your Risk.

[69] Griffiths D, Sturm J (2011). Epidemiology and etiology of young stroke. Stroke Res Treat.

[70] Kamel H, Navi BB, Sriram N (2014). Risk of a thrombotic event after the 6-week postpartum period. N Engl J Med.

[71] Gargano JW, Wehner S, Reeves MJ (2009). Do presenting symptoms explain sex differences in emergency department delays among patients with acute stroke?. Stroke.

[72] Lisabeth LD, Brown DL, Hughes R (2009). Acute stroke symptoms: comparing women and men. Stroke.

[73] Labiche LA, Chan W, Saldin KR (2002). Sex and acute stroke presentation. Ann Emerg Med.

[74] Jerath NU, Reddy C, Freeman D (2011). Gender differences in presenting signs and symptoms of acute ischemic stroke: a population-based study. Gend Med.

[75] Stuart-Shor EM, Wellenius GA, DelloIacono DM (2009). Gender differences in presenting and prodromal stroke symptoms. Stroke.

[76] Mandelzweig L, Goldbourt U, Boyko V (2006). Perceptual, social, and behavioral factors associated with delays in seeking medical care in patients with symptoms of acute stroke. Stroke.

[77] Reeves MJ, Fonarow GC, Zhao X (2009). Quality of care in women with ischemic stroke in the GWTG program. Stroke.

[78] Forster A, Gass A, Kern R (2009). Gender differences in acute ischemic stroke: etiology, stroke patterns and response to thrombolysis. Stroke.

[79] Tsivgoulis G, Alexandrov AV, Chang J (2011). Safety and outcomes of intravenous thrombolysis in stroke mimics: a 6-year, single-care center study and a pooled analysis of reported series. Stroke.

[80] Zinkstok SM, Engelter ST, Gensicke H (2013). Safety of thrombolysis in stroke mimics: results from a multicenter cohort study. Stroke.

[81] Arch AE, Weisman DC, Coca S (2016). Missed Ischemic Stroke Diagnosis in the Emergency Department by Emergency Medicine and Neurology Services. Stroke.

[82] Kent DM, Price LL, Ringleb P (2005). Sex-based differences in response to recombinant tissue plasminogen activator in acute ischemic stroke: a pooled analysis of randomized clinical trials. Stroke.

[83] Hill MD, Kent DM, Hinchey J (2006). Sex-based differences in the effect of intra-arterial treatment of stroke: analysis of the PROACT-2 study. Stroke.

[84] Madsen TE, Seigel TA, Mackenzie RS (2014). Gender differences in neurologic emergencies part I: a consensus summary and research agenda on cerebrovascular disease. Acad Emerg Med.

[85] Ahnstedt H, McCullough LD, Cipolla MJ (2016). The Importance of Considering Sex Differences in Translational Stroke Research. Transl Stroke Res.

[86] Towfighi A, Markovic D, Ovbiagele B (2013). Sex differences in revascularization interventions after acute ischemic stroke. J Stroke Cerebrovasc Dis.

[87] Tarasow E, Abdulwahed Saleh Ali A, Lewszuk A (2007). Measurements of the middle cerebral artery in digital subtraction angiography and MR angiography. Med Sci Monit.

[88] Reeves MJ, Bushnell CD, Howard G (2008). Sex differences in stroke: epidemiology, clinical presentation, medical care, and outcomes. Lancet Neurol.

[89] Tate J, Bushnell C (2011). Pregnancy and stroke risk in women. Womens Health.

[90] Selim MH, Molina CA (2013). The use of tissue plasminogen-activator in pregnancy: a taboo treatment or a time to think out of the box. Stroke.

[91] Broderick JP (2013). Should intravenous thrombolysis be considered the first option in pregnant women?. Stroke.

[92] Nicolson TJ, Mellor HR, Roberts RR (2010). Gender differences in drug toxicity. Trends Pharmacol Sci.

[93] Chen ML, Lee SC, Ng MJ (2000). Pharmacokinetic analysis of bioequivalence trials: implications for sex-related issues in clinical pharmacology and biopharmaceutics. Clin Pharmacol Ther.

[94] Tran C, Knowles SR, Liu BA (1998). Gender differences in adverse drug reactions. J Clin Pharmacol.

[95] Soldin OP, Chung SH, Mattison DR (2011). Sex differences in drug disposition. J Biomed Biotechnol.

[96] Schwartz J (2006). The Current State of Knowledge on Age, Sex, and Their Interactions in Clinical Pharmacology. Clin Pharm & Therap.

[97] Office, U. S. G. A. (2001). Drug Safety: Most Drugs Withdrawn in Recent Years Had Greater Health Risks for Women, GAO-01-286R Drugs Withdrawn From Market.

[98] Gandhi M, Aweeka F, Greenblatt RM (2004). Sex differences in pharmacokinetics and pharmacodynamics. Annu Rev Pharmacol Toxicol.

[99] Jovanovic H, Lundberg J, Karlsson P (2008). Sex differences in the serotonin 1A receptor and serotonin transporter binding in the human brain measured by PET. Neuroimage.

[100] Schwartz J (2006). The Current State of Knowledge on Age, Sex, and Their Interactions in Clinical Pharmacology. Clin Pharm & Therap.

[101] Teff KL, Alavi A, Chen J (1999). Muscarinic blockade inhibits gastric emptying of mixed-nutrient meal: effects of weight and gender. Am J Physiol.

[102] Stephen AM, Wiggins HS, Englyst HN (1986). The effect of age, sex and level of intake of dietary fibre from wheat on large-bowel function in thirty healthy subjects. Br J Nutr.

[103] Campesi IFM, Franconi F (2012). Handbook of Experimental Pharmacology.

[104] Fillingim RB, King CD, Ribeiro-Dasilva (2009). Sex, gender, and pain: a review of recent clinical and experimental findings. J Pain.

[105] Hreiche R, Morissette P, Turgeon J (2008). Drug-induced long QT syndrome in women: review of current evidence and remaining gaps. Gend Med.

[106] Milou-Daniel Drici NC (2001). Is Gender a Risk Factor for Adverse Drug Reactions? The Example of Drug-Induced Long QT Syndrome. Drug Safety.

[107] Gupta A, Lawrence AT, Krishnan K (2007). Current concepts in the mechanisms and management of drug-induced QT prolongation and torsade de pointes. Am Heart J.

[108] Rodriguez I, Kilborn MJ, Liu XK (2001). Drug-induced QT prolongation in women during the menstrual cycle. JAMA.

[109] Coker SJ (2008). Drugs for men and women - how important is gender as a risk factor for TdP?. Pharmacol Ther.

[110] Jonsson MK, Vos MA, Duker G, Demolombe S, van Veen TA (2010). Gender disparity in cardiac electrophysiology: implications for cardiac safety pharmacology. Pharmacol Ther.

[111] (2014). Equality, National Center for Transgender Quality, Transgender Terminology, 1325 Massachusetts Avenue NW, Suite 700, Washington DC.

