# Supplementary material for: Sex as a Biological Variable in Emergency Medicine Research and Clinical Practice: A Brief Narrative Review
Source: West J Emerg Med. 2017 Oct 6;18(6):1079–90. doi: 10.5811/westjem.2017.8.34997 (PMC5654878; doi:10.5811/westjem.2017.8.34997)
Supplement: Supplementary file 1 [file wjem-18-1079-s001.docx]

**Appendix.** Definitions pertaining to sex and gender

Adapted from National Center for Transgender Equality^[[1]](#endnote-1)^

| **Sex** | Classification of a person by chromosomal complement |
| --- | --- |
| **Gender** | A person’s self-representation as male or female. Rooted in biology and shaped by environment and experience. |
| **Gender Identity** | An individual’s internal sense of being male, female, or something else. Since gender is internal, one’s gender identity is not necessarily visible to others. |
| **Transsexual** | An older term for people whose gender identity is different from their assigned sex at birth who seeks to transition from male to female or female to male. Many do not prefer this term because it is thought to sound overly clinical. |
| **Transgender** | A term for people whose gender identity, expression or behavior is different from those typically associated with their assigned sex at birth |
| **Transgender Women** | A term for an individual of male sex who currently identifies as female gender |
| **Transgender Man** | A term for an individual of female sex who currently identifies as a male gender. |
| **Cisgender** | Gender identity that matches their assigned sex |
| **Intersex** | Reproductive anatomy and/or chromosome pattern that does not fit typical definitions of male or female. Also known as differences of sex development. Examples include Klinefelter (XXY), Turner (XO), Sry Male (XX with a y gene on X chromosome), Androgen Insensitivity or medical conditions such as Congenital Adrenal Hyperplasia. |

1. Equality, National Center for Transgender Quality, Transgender Terminology, 1325 Massachusetts Avenue NW, Suite 700, Washington DC, 2014. Accessed Aug 30, 2016. [↑](#endnote-ref-1)
